# Precision Nutrition and Omega-3 Polyunsaturated Fatty Acids: A Case for Personalized Supplementation Approaches for the Prevention and Management of Human Diseases

**DOI:** 10.3390/nu9111165

**Published:** 2017-10-25

**Authors:** Floyd H. Chilton, Rahul Dutta, Lindsay M. Reynolds, Susan Sergeant, Rasika A. Mathias, Michael C. Seeds

**Affiliations:** 1 Department of Physiology and Pharmacology, Wake Forest School of Medicine, Winston-Salem, NC 27157, USA; 2 Department of Urology, Wake Forest School of Medicine, Winston-Salem, NC 27157, USA; rdutta@wakehealth.edu; 3 Department of Epidemiology and Prevention, Wake Forest School of Medicine, Winston-Salem, NC 27157, USA; lireynol@wakehealth.edu; 4 Department of Biochemistry, Wake Forest School of Medicine, Winston-Salem, NC 27157, USA; ssergean@wakehealth.edu; 5 GeneSTAR Research Program, General Internal Medicine, Johns Hopkins University School of Medicine, Baltimore, MD 21224, USA; rmathias@jhmi.edu; 6 Department of Internal Medicine, Section on Molecular Medicine, Wake Forest School of Medicine, Winston-Salem, NC 27157, USA; mseeds@wakehealth.edu

**Keywords:** omega-3 fatty acids, polyunsaturated fatty acids, gene-diet interaction, human disease, inflammation, fatty acid desaturase genes, arachidonic acid, eicosanoids, endocannabinoids

## Abstract

Background: Dietary essential omega-6 (*n*-6) and omega-3 (*n*-3) 18 carbon (18C-) polyunsaturated fatty acids (PUFA), linoleic acid (LA) and α-linolenic acid (ALA), can be converted (utilizing desaturase and elongase enzymes encoded by *FADS* and *ELOVL* genes) to biologically-active long chain (LC; >20)-PUFAs by numerous cells and tissues. These *n*-6 and *n*-3 LC-PUFAs and their metabolites (ex, eicosanoids and endocannabinoids) play critical signaling and structural roles in almost all physiologic and pathophysiologic processes. Methods: This review summarizes: (1) the biosynthesis, metabolism and roles of LC-PUFAs; (2) the potential impact of rapidly altering the intake of dietary LA and ALA; (3) the genetics and evolution of LC-PUFA biosynthesis; (4) Gene–diet interactions that may lead to excess levels of *n*-6 LC-PUFAs and deficiencies of *n*-3 LC-PUFAs; and (5) opportunities for precision nutrition approaches to personalize *n*-3 LC-PUFA supplementation for individuals and populations. Conclusions: The rapid nature of transitions in 18C-PUFA exposure together with the genetic variation in the LC-PUFA biosynthetic pathway found in different populations make mal-adaptations a likely outcome of our current nutritional environment. Understanding this genetic variation in the context of 18C-PUFA dietary exposure should enable the development of individualized *n*-3 LC-PUFA supplementation regimens to prevent and manage human disease.

## 1. Introduction

Modern humans emerged from Africa ~200,000 years ago and spread across the earth over the past 100,000 years. During this time, available food sources created evolutionary pressure that drove genetic architecture which allowed our species to adapt, survive and proliferate; this was coupled with a rapid expansion in brain size (particularly gray matter in the cerebral cortex) and, with it, advancements in human intelligence, socialization, and innovation.

The modern Western diet (MWD) has dramatically changed the nutritional content of ingested foods in developed countries, and given the rapid nature of these nutritional transitions, mal-adaptations and related human diseases are a likely outcome of our current nutritional environment [1,2]. For example, up to 72% of dietary calories consumed presently in the MWD did not exist in hunter-gatherer diets [3]. Changes in food type (quality) and quantity in the MWD have been largely driven by technological changes in food production and processing to provide high-calorie and appealing food (high in sugars, refined grains and oils) to large urban populations [1,4]. These have led to detrimental shifts in nutrient metabolism leading to gene-diet interactions responsible for more obesity and localized and systemic inflammation [2]. In turn, this inflammation contributes to the pathogenesis of a variety of disease states, including cardiovascular disease, diabetes and insulin resistance, cancer, autoimmunity, hypersensitivity disorders such as asthma and allergies, chronic joint disease, skin and digestive disorders, dementia and Alzheimer’s disease [5,6,7,8,9,10,11,12,13,14]. As challenging as these changes are for overall populations of developed countries such as the US, they are more negative for certain populations and ethnic groups [15,16,17,18,19,20], in whom a disproportionate burden of preventable disease, death, and disability now exists. However, the emergence of the field of precision nutrition that factors in individual- and population-based genetic variability in the context of human diets offers the promise to provide more specific and individualized dietary and supplement interventions that may prevent and mitigate many of the pro-inflammatory effects of the MWD [21].

With regard to fatty acid (FA) intake, there has been marked shift (due largely to recommendations from health agencies) to reduce levels of saturated fatty acids and replace them with polyunsaturated fatty acids (PUFAs) in an attempt to lower serum total cholesterol and LDL lipoproteins [22,23]. From a practical perspective, this meant a replacement of sources of saturated fat such as lard and butter with PUFA-containing vegetable oils (soybean, corn, and canola oils, as well as margarine and shortenings), which are rich in the 18 carbon (18C) omega-6 (*n*-6) PUFA linoleic acid (18:2*n*-6, LA) and poor in both the omega-3 (*n*-3) 18C-PUFA, α-linolenic acid (18:3*n*-3, ALA) and monounsaturated fatty acids. In fact, it has been estimated that soybean oil consumption alone (which contains 58 g LA/100 g oil) increased >1000-fold from 1909 to 1999 and now contributes to ~7% of daily energy of the MWD [1]. Over time, this progressive increase in the ingestion of vegetable oils has led to a 3-fold increase (to 6–8% energy) in dietary LA content of the MWD [1,3,24,25,26], as well as an estimated 40% reduction in total *n*-3 long chain (≥20 carbon; LC-) PUFA levels, and a large shift in the ratio of dietary *n*-6/*n*-3 C18 PUFAs consumed from ~5:1 to >10:1 [1,27].

The objectives of this review are first to point out how lifestyle variables and specifically our current dietary PUFA exposure together with ancestral-based genetic variation in the LC-PUFA biosynthetic pathway gives rise to distinct molecular profiles (levels of LC-PUFAs, LC-PUFA metabolites, inflammatory and other disease biomarkers) that enhance disease risk for certain individuals and populations. These gene-diet interactions may be particularly important to health in western countries as dietary *n*-6 and *n*-3 18C PUFAs comprise almost 10% of daily calories in the MWD. The second objective of the review is to describe how an understanding of PUFA-based gene-diet interactions can provide a scientific basis for the development of specific dietary and supplement strategies with *n*-3 LC-PUFAs to prevent and manage human diseases.

## 2. Long Chain Polyunsaturated Fatty Acid Biosynthesis and Biological Activities

**Figure 1:**
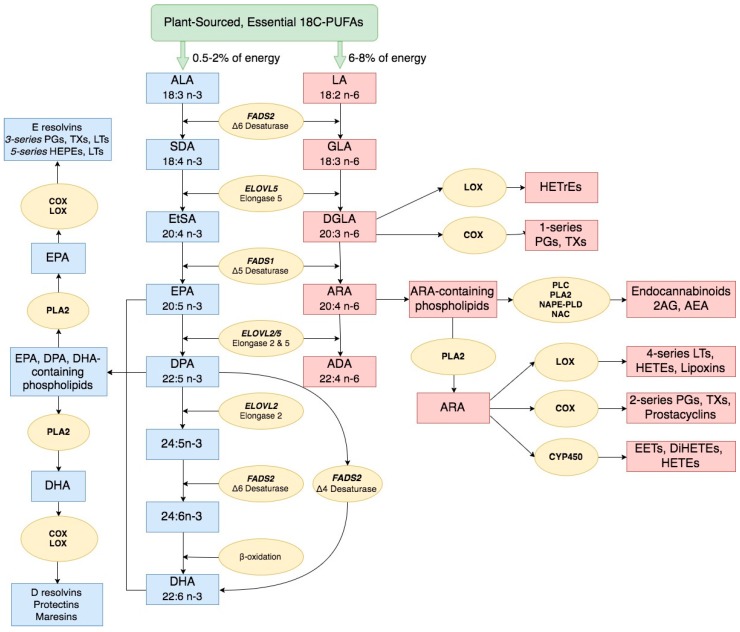
Polyunsaturated fatty acid biosynthesis. *n*-3 and *n*-6 LC-PUFA are synthesized from dietary intake of essential fatty acids ALA and LA, respectively, through a series of enzymatic desaturation (*FADS2* and *FADS1*) and elongation (*ELOVL2* and *ELOVL5*) steps. This pathway gives rise to primary *n*-3 LC-PUFAs and *n*-6 LC-PUFAs such as EPA, DPA, DHA and ARA. These LC-PUFAs (as free fatty acids or complex lipids) and their metabolites impact a wide ranges of physiologic and pathophysiologic processes. Abbreviations: *FADS1/2*, fatty acid desaturase 1/2; *ELOVL 2/5*, fatty acid elongase 2/5; ALA, α-linolenic acid; SDA, stearidonic acid; EtSA, eicosatetraenoic acid; EPA, eicosapentaenoic acid; DPA, docosapentaenoic acid; DHA, docosahexaenoic acid; LA, linoleic acid; GLA, γ-linolenic acid; DGLA, dihomo-γ-linolenic acid; ARA, arachidonic acid; ADA, adrenic acid; PG, prostaglandin; TX, thromboxane; LT, leukotriene; HEPE, hydroxyeicosapentaenoic acid; HETrE, hydroxyeicosatrienoic acid, HETE, hydroxyeicosatetraenoic acid; DiHETE, dihydroxyeicosatetraenoic acid; EET, epoxyeicosatetraenoic acid; 2AG, 2-arachidonoylglycerol; AEA, arachidonoyl ethanolamide/anandamide.

From the work of George and Mildred Burr almost 100 years ago [28,29], which was extended by the studies of Ralph Holman [30,31], it became clear that *n*-3 and *n*-6 18C-PUFAs were essential for human health. Furthermore, these 18C-PUFAs originated from the diet and were not synthesized from acetyl and malonyl CoA ester condensations catalyzed by fatty acid synthase. The two essential dietary PUFAs of shortest (18C) chain length, ALA and LA, are the key substrates that enter the biosynthetic pathways leading to biologically-active *n*-3 and *n*-6 LC-PUFAs, respectively. Figure 1 highlights the LC-PUFA biosynthetic pathway and genes known to encode for enzymes that play key roles in the two parallel and competing pathways that synthesize *n*-3 and *n*-6 LC-PUFAs. Two desaturation enzymes encoded by fatty acid desaturase 1 and 2 (*FADS1* and *FADS2*) and one elongation enzyme encoded by *ELOVL5* synthesize eicosapentaenoic acid (20:5*n*-3, EPA) and arachidonic acid (20:4*n*-6, ARA) from ALA and LA, respectively [32,33,34,35,36]. The *n*-3 LC-PUFA, docosapentaenoic acid (22:5*n*-3; DPA) and the *n*-6 LC-PUFA, adrenic acid (22:4*n*-6; ADA) can be generated from EPA and ARA, respectively, using an additional elongation enzyme (encoded by *ELVOL 5/2*), and finally docosahexaenoic acid (22:6*n*-3; DHA) can be produced from DPA with a ∆-4 desaturation enzyme also encoded by *FADS2* [37]. EPA may also be converted to DHA utilizing three additional biosynthetic steps (2 elongation, 1 desaturation and 1 β-oxidation). Smaller quantities of LC-PUFAs can be obtained directly from the diet. For example, preformed ARA is found in organ meats, eggs, poultry, and fish, and various types of seafood such as cold-water fish are rich in preformed *n*-3 LC-PUFAs, EPA, DPA and DHA [26].

Once formed, LC-PUFAs have many roles as free fatty acids and esterified in complex lipids (Figure 1). These include biophysical properties essential for proper plasma membrane function, energy production by β-oxidation and specific biochemical roles as precursors of bioactive lipids [38,39]. For example, in the central nervous system, the *n*-3 LC-PUFA DHA is the most abundant FA in complex lipids constituting approximately 50% of the weight of neuronal plasma membranes. Its membrane status and signaling capacity directly impact brain development and function via several mechanisms, including maintaining membrane integrity, neurotransmission, neurogenesis, membrane receptor function and signal transduction [38,40,41,42,43,44].

Importantly, both *n*-6 and *n*-3 LC-PUFAs are also converted to a diverse family of metabolites including multiple forms of prostaglandins, thromboxanes, hydroxyeicosatetraenoic acids, epoxyeicosatrienoic acids, leukotrienes, lipoxins, resolvins, protectins, maresins and endocannabinoids (Figure 1) [45,46,47,48,49,50,51,52]. LC-PUFAs and their metabolites, along with their cellular receptors, are present in practically all cells and tissues of the body and act as potent signaling molecules that impact a wide range of physiologic and pathophysiologic processes [45,46,47,48,49,50,51,52].

Most evidence to date indicates that *n*-6 and *n*-3 LC-PUFAs and their metabolic products have not only different, but often opposing effects on immunity and inflammation [53,54,55,56,57]. In general, *n*-6 LC-PUFA metabolites and particularly ARA act as local hormones to promote acute and chronic inflammation [46,51,52]. In contrast to ARA, *n*-3 LC-PUFAs, such as EPA, DPA, and DHA, can be metabolized to anti-inflammatory mediators that have “pro-resolution” properties [47,58]. An exception to this principle are the ARA-derived lipoxins that exert anti-inflammatory, pro-resolution bioactions [59].

Over the past 20 years, one of the most fascinating areas of science has been the discovery of the pleiotropic effects of the endocannabinoid system [60,61,62]. Endocannabinoids have been shown to be complex lipids (such as 2-arachidonoyl glycerol and arachidonyl ethanolamide) derived from the *n*-6 LC-PUFA, ARA [60,61,62]. More recent studies have demonstrated that *n*-3 LC-PUFA derivatives of endocannabinoids also exist [63,64,65]. Endocannabinoid action via cannabinoid 1 and 2 receptors impacts a wide range of biological functions including energy balance and metabolism, mood, memory, sleep, reproduction, thermoregulation and immune function [63,64,65,66,67,68,69,70,71,72,73,74,75]. Endocannabinoids can also be metabolized by cyclooxygenases, lipoygenases, and p450 epoxygenases to form other biologically-active complex lipids [65,76,77].

It is clear from the aforementioned studies that *n*-6 and *n*-3 LC-PUFAs and their metabolites have structural and/or signaling roles throughout the human body (Figure 1). Additionally, maintaining a proper balance of *n*-6 and *n*-3 LC-PUFAs and their metabolites is critical to homeostasis in virtually every physiologic system. Consequently, environmental and genetic mechanisms that influence their levels and balance will impact human health and disease.

## 3. Impact of Dietary Linoleic Acid and α-Linolenic Acid Levels on *n*-3 LC-PUFA Biosynthesis

Several studies have warned against health and disease outcomes that could result from radical increases of dietary LA in the MWD in such a short period of time [26,78,79]. Given the shared enzymatic steps involved in the processing of LA and ALA, these *n*-6 and *n*-3 18C-PUFAs and their metabolic intermediates compete with each other in the liver and other tissues as substrates for synthetic enzymatic reactions that produce LC-PUFAs [80,81]. Additionally, there is an overall limited capacity of 18C-PUFAs that can be converted to LC-PUFAs [53]. As discussed in detail below, this biosynthetic limit in capacity is highly impacted at an individual level by genetic variation in the LC-PUFA biosynthetic pathway. Consequently, a dramatic increase in LA in the MWD observed over the past 75 years together with competition between *n*-6 and *n*-3 substrates within the pathway has been shown in animal models and humans to shift the pathway toward the biosynthesis of high levels *n*-6 LC-PUFAs and away from *n*-3 LC-PUFAs [53,82,83,84,85,86,87]. In 1992, Lands and colleagues described non-linear interactions between LA and ALA in forming LC-PUFAs utilizing a hyperbolic equation that fit for rats, mice and humans [53]. The equation points out the limitation of generating *n*-3 LC-PUFAs when *n*-3 ALA is ingested together with several-fold greater amounts of *n*-6 LA as is the case with the MWD. Wood and colleagues reviewed human studies that examined the effect of altering LA and ALA on *n*-6 and *n*-3 LC-PUFA biosynthesis and concluded that it is possible to increase *n*-3 LC-PUFAs by reducing LA or increasing ALA intake in humans [82]. However, LA levels need to be reduced to <2.5% energy before levels of DHA can be increased. Again, typical LA levels in the MWD reside between 6–8% energy; consequently, high levels of LA in the MWD would be predicted to markedly reduce, not increase DHA. In fact, it has been estimated that LA concentrations in the MWD have decreased the omega-3 index by 41%, from 6.51 to 3.84 [1].

A 1997 paper by Okuyama and colleagues made a compelling case that excess LA and the increase in the LA/ALA ratio as a result of moving away from traditional diets led to ‘Omega-3 Deficiency Syndrome’ in the elderly in Japan [84]. The paper summarized the “evidence which indicates that increased dietary LA and relative *n*-3 deficiency are major risk factors for western-type cancers, cardiovascular and cerebrovascular diseases and also for allergic hyper-reactivity.” They also suggest that *n*-3 LC-PUFAs deficiency created by excess LA and LA/ALA ratios in the MWD affects human behavior patterns in industrialized countries. Certainly these assertions are supported by a large body of scientific literature in both animal models and human studies discussed throughout this review.

## 4. The Genetics and Evolution of LC-PUFA Biosynthesis

Through a better understanding of genetic variation associated with the utilization of specific nutrients, precision nutrition approaches offer the potential to predict the physiological and pathological consequences of the interaction of individual genetic differences and diet to prevent and/or manage adverse outcomes. Until recently, it was assumed that the metabolic capacity of the LC-PUFA biosynthetic pathway was limited and fairly uniform in all humans. This premise was supported by metabolic studies in European ancestry populations, which suggest that only a small proportion of ingested dietary 18C PUFAs (typically 2–3% energy) are converted into LC-PUFAs [81,88,89,90]. However, studies over the past decade have demonstrated common genetic and epigenetic variation in genes (including *FADS1*, *FADS2*, *ELOVL5* and *ELOVL2*) throughout the LC-PUFA biosynthetic pathway are highly associated with the levels of LC-PUFAs produced in human circulation, cells and tissues [91,92,93,94,95,96,97,98,99,100,101,102,103,104,105,106,107,108,109,110,111,112,113,114,115,116]. This body of evidence has challenged the concept that LC-PUFA biosynthesis from 18C-PUFAs is uniform among individuals and populations.

**Figure 2:**
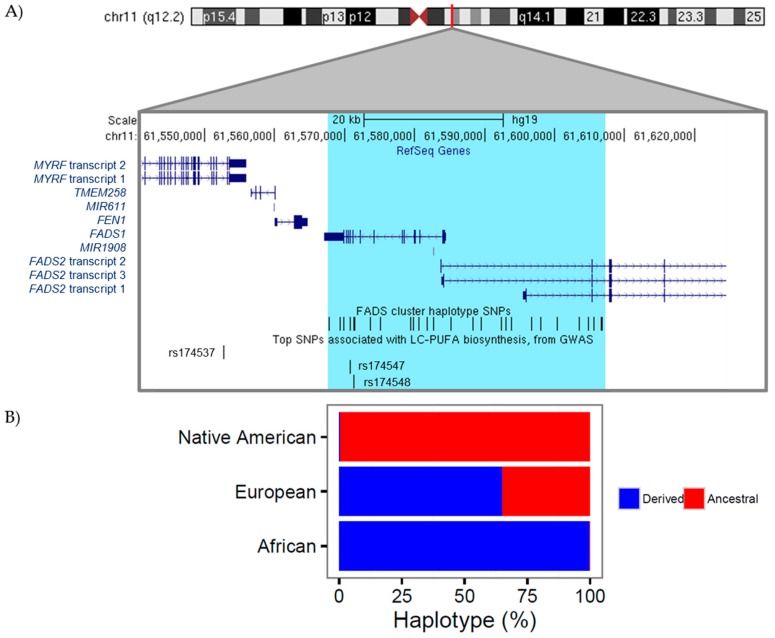
Genetic variation near the *FADS* gene cluster. (**A**) A expanded depiction of the *FADS* gene cluster on chromosome 11 illustrates the genomic location (build hg19) of: genes in this region (shown in dark blue, from RefSeq), the *FADS* cluster haplotype region and single nucleotide polymorphisms (SNPs) (region highlighted in light blue with SNPs shown as black vertical bars), and three individual SNPs identified as the most significantly associated genetic variants genome-wide with LC-PUFA levels (rs174537, rs174547, and rs174548); (**B**) The observed percentage of derived vs. ancestral *FADS* cluster haplotype varies by ethnicity.

The desaturase enzymes within the pathway, encoded by the two genes (*FADS1*, *FADS2*) in the *FADS* cluster region (chr11: 61,540,615–61,664,170) have long been recognized as the rate-limiting steps in the conversion of 18C-PUFAs to LC-PUFA (Figure 1 and Figure 2). Our laboratory initially demonstrated that there are marked differences between African and European ancestry populations in the circulating levels of *n*-6 and *n*-3 LC-PUFAs [100,106]. This work also showed that ~80% of African Americans carry two copies of *FADS* alleles associated with more efficient biosynthesis of LC-PUFAs, compared to only ~45% of European Americans and these genetic differences explained a large proportion of the variability in LC-PUFA levels between African and European Americans. Numerous other studies have also revealed strong genetic influences within the *FADS* cluster region on circulating, cellular and tissue levels of LC-PUFAs [2,36,117]. This region of association comprises a linkage disequilibrium (LD) block covering the promoter regions of both *FADS1* and *FADS2* (Figure 2)*.* Importantly, the derived haplotype that includes numerous genetic variants with common allele frequencies (when compared to the ancestral haplotype) is associated with higher levels of LC-PUFAs and an increased efficiency (as determined by product to precursor ratios within the LC-PUFA biosynthetic pathway) by which LC-PUFAs are synthesized [117]. One of the most surprising aspects of these genetic studies is the observation that there are dramatic differences in the frequencies of the ancestral and derived haplotypes and thus the efficiency of LC-PUFA biosynthesis among diverse global populations. For example, the ancestral haplotype is most common (97%) in Native Americans and virtually absent in Africa, suggesting that Native Americans and individuals of Native American ancestry have a more limited capacity than Africans to synthesize LC-PUFAs [118]. The derived haplotype is observed at varying frequencies (25–50%) in Europe and East Asia [117,119]. As described in detail below, extensive evolution of the *FADS* cluster and thus changes in the efficiency of LC-PUFA biosynthesis took place as early humans adapted to local environments as they moved from Africa to the Americas. These are reflected in the dramatic differences in the *FADS* haplotype frequencies and LC-PUFA biosynthetic efficiencies observed in diverse modern populations.

Numerous individual genetic variants within the *FADS* cluster (as illustrated in Figure 2) have been identified by genome-wide association studies (GWAS) to be highly associated with LC-PUFA levels as well a wide variety of important molecular and clinical phenotypes. For instance, GWAS of plasma *n*-3 and *n*-6 LC-PUFA levels in Italian, European, and Chinese populations have identified single nucleotide polymorphisms (SNPs) such as rs174537 and rs174547 located near *FADS1,* which are the strongest signals genome-wide associating with levels of *n*-3 and *n*-6 LC-PUFAs such as ARA and EPA [94,101,120,121,122]. Genetic variants in the *FADS* cluster are also associated with numerous human phenotypes including inflammatory and cardiovascular disorders, blood lipid levels including low-density lipoprotein (LDL) and triglyceride levels, coronary artery disease, insulin resistance, perinatal depression, atopic diseases, attention/hyperactivity, intelligence and memory in children [36,92,96,99,102,105,109,111,116,123,124,125,126,127,128]. The strong associations between genetic variation in the *FADS* cluster, LC-PUFA levels, and important clinical phenotypes support the concept that genetically-induced alterations in LC-PUFA levels may play important roles in several human diseases. However, due to the high degree of LD observed between genetic variants in this region, it is difficult to determine which variants have a casual role in altering *FADS* activity thus the efficiency of LC-PUFA biosynthesis.

An important question from this work is: why are there such distinct ancestral-based genetic variation within the *FADS* cluster? It is important to understand that nutrients and genomes interact reciprocally. As described above, genetic variation confers differences in nutrient utilization. However, changes in nutrient exposure throughout human development also created adaptive pressures that led to selection of genetic variation that better fit nutritional environments. Our laboratory initially examined evolutionary forces shaping patterns of variation in the *FADS* cluster by examining geographically diverse populations representing 14 populations and focused on a 300 kb region centered on the *FADS* loci [119]. This work confirmed that there are marked global differences in allele frequencies of variants in the *FADS* gene cluster that are strongly associated with the efficiency of conversion of LA and ALA to ARA and DHA, respectively. This study also provided evidence that alleles associated with LC-PUFA biosynthesis were driven to near fixation in African populations by positive selection ~85,000 years ago. The selection of these *FADS* variants would have enhanced LC-PUFA synthesis from plant-sourced 18C-PUFAs. We postulate that this enhanced the capacity to synthesize LC-PUFAs and particularly *n*-3 LC-PUFAs such as DHA, and was therefore an important advantage that would have facilitated the movement of humans away from marine sources of LC-PUFAs in isolated geographic regions and concomitant rapid expansion and migration throughout the African continent 60,000–80,000 years ago. That same time, Ameur and colleagues described the *FADS* haplotype patterns (ancestral and derived), with the derived haplotype found in Africa, that were associated with more efficient conversion of 18C-PUFAs into LC-PUFAs [117].

However, it was unclear from these initial papers: (1) why human populations migrating out of Africa appeared to carry the ancestral haplotype; (2) why Eurasian populations are polymorphic; and (3) why the ancestral haplotype is at near fixation in Native American populations. Fumagalli and colleagues provided an important clue to this puzzle by carrying out a genome-wide scan for positive selection in the Greenlandic Inuit and showing that genetic variation in the *FADS* cluster to be the strongest signatures for cold adaptation [129]. Interestingly, the two most highly differentiated SNPs (rs7115739 and rs174570) were associated with lower levels of LC-PUFAs and higher levels of 18C-PUFAs precursors. It was also demonstrated that these *FADS* cluster alleles were associated with a decrease in weight, height, fasting serum insulin, and fasting serum LDL cholesterol [129]. These investigators posited that the challenging environmental conditions of the Arctic likely imposed strong selective pressures on the Inuit and their ancestors and these physical and molecular phenotypes were important to cold adaptation. However, the PUFA-related molecular mechanism responsible for physical phenotypes such as weight and height are not yet understood. Very recently, we have demonstrated that the ancestral haplotype frequency is also correlated to Siberian populations’ geographic location, further suggesting the ancestral haplotype’s role in cold weather adaptation [130]. Additionally, this likely explains the high haplotype frequency of the ancestral haplotype within Native American populations [118,130].

There are several other recently published papers which have focused on the role that evolution of the *FADS* cluster played in the capacity of humans to adapt to varying global nutritional environments containing fluctuating levels of LC-PUFAs. For example, Mathieson and colleague carried out a genome-wide scan for positive selection from ancient and present-day European genomes and demonstrated strong selection for derived alleles over the past 4000 years [131]. Kothapalli and colleagues studied genomes from populations in South Asia and showed positive selection for an indel in *FADS2* that is associated with more efficient LC-PUFA biosynthesis [132]. They suggest that this was an important adaptation as populations moved to more vegetarian diets with low levels of dietary LC-PUFAs [132]. Buckley and colleagues compared *FADS* sequencing data from present day to the Bronze Age and concluded that selection patterns in Europeans were driven by changes in dietary fat composition and specifically LC-PUFA levels following the transition to agriculture [133]. Taken together, all of these studies reflect the evolutionary importance for humans to regulate LC-PUFA biosynthesis. The complex interactions among local selective pressures in diverse local environments along global human migration patterns appears to have given rise to the global variation in frequencies of the derived and ancestral haplotypes that are observed today.

## 5. Anatomy of *n*-6 LC-PUFA Excesses and *n*-3 LC-PUFA Deficiencies

**Figure 3:**
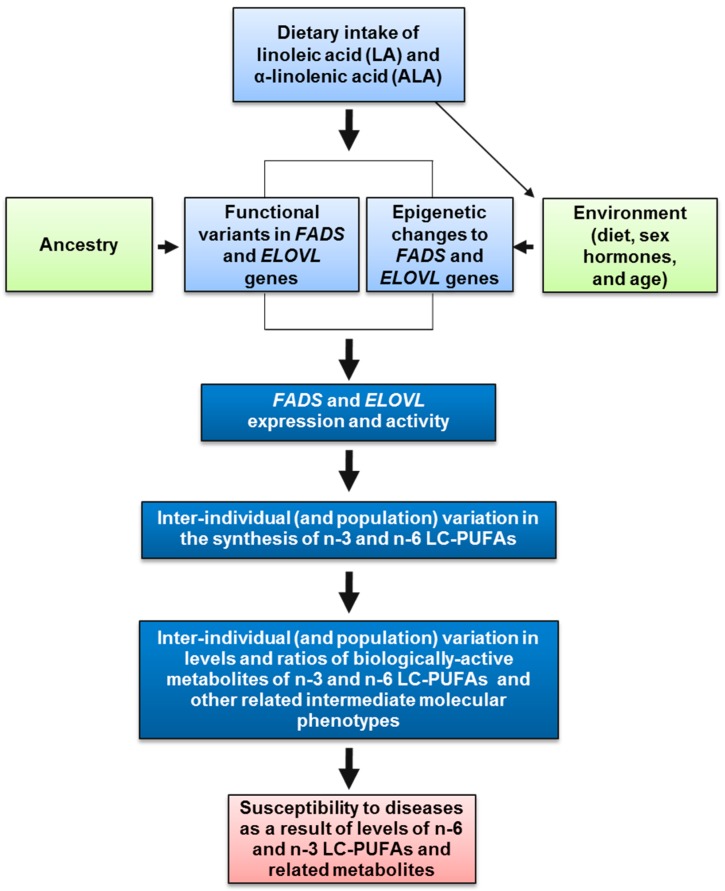
Anatomy of gene-diet interactions leading to *n*-6 LC-PUFA excesses and *n*-3 LC-PUFA deficiencies. Dietary intake of *n*-3 and *n*-6 18C-PUFAs, ALA and LA, respectively, interact with *FADS* or *ELOVL* genetic and epigenetic variation (that impacts *FADS* or *ELOVL* expression or resultant activity) to determine circulating and cellular levels of *n*-3 and *n*-6 LC-PUFAs. These interactions can result in an unhealthy balance of LC-PUFAs, with excess levels of *n*-6 LC-PUFAs or deficiencies of *n*-3 LC-PUFAs.

As outlined throughout this review, there are several components of the MWD and the diverse genetics of LC-PUFA biosynthesis among different human populations that could combine to create harmful gene-diet interactions, which in turn would impact levels of *n*-6 and *n*-3 LC-PUFAs, their metabolites and ultimately human disease (Figure 3). First, gene-diet interactions can arise when there is a major change in the exposure of a nutrient that is utilized by an important metabolic pathway. As discussed above, following recommendations to replace dietary saturated fatty acids with PUFAs, food production companies began replacing the saturated fatty acids, largely with *n*-6 18C-PUFAs, and this led to a dramatic increase (~3 fold) in the ingestion of LA. In contrast, the ingestion of the dietary *n*-3 18C-PUFA, ALA, has remained relatively constant [1]. This resulted in a significant change in not only LA exposure but also the ratio of dietary LA to ALA that enters the LC-PUFA biosynthetic pathway. As discussed above, LA and its *n*-6 metabolites directly compete with ALA and its *n*-3 metabolites in the synthesis of LC-PUFAs, and there is a limited capacity of the pathway to produce LC-PUFAs. Consequently, the ratio of LA to ALA has been altered by increasing dietary LA (to 6–8% of energy) and this dietary modification has shifted the pathway toward the biosynthesis of higher levels *n*-6 LC-PUFAs and away from *n*-3 LC-PUFAs.

A second component of potentially harmful gene-diet interactions is exemplified in some individuals or human populations that have a greater genetic capacity to more efficiently utilize/metabolize a specific nutrient than others. A well-recognized example of this situation are the variants near the *LCT* locus that codes for the lactase enzyme, which metabolizes lactose in milk [134,135,136]. Cattle domestication ~10,000 years ago induced strong selection to be able to utilize lactose, the primary carbohydrate in milk, as adults. In most humans, levels of the lactase enzyme decreases after weaning, but certain populations that traditionally depended on milk have variants near the *LTC* locus associated with high levels of lactase and thus retain the capacity to utilize lactose into adulthood.

**Figure 4:**
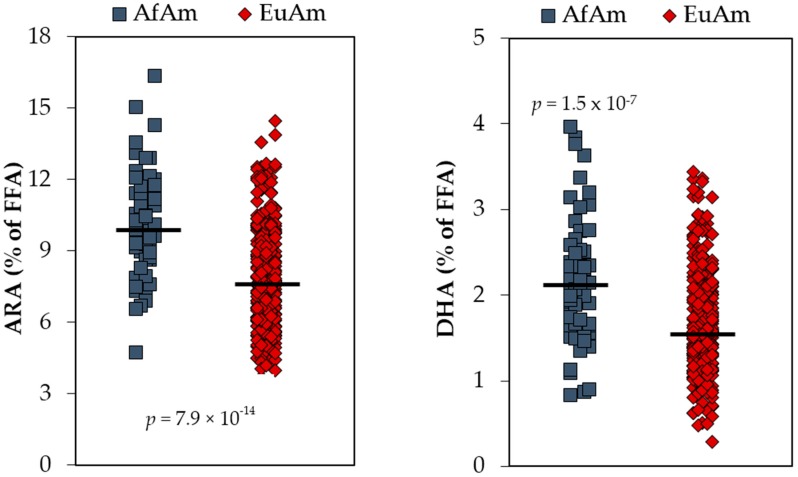
Serum Levels of ARA and DHA in African Americans (AfAm) and European Americans (EuAm). Both *n*-6 and *n*-3 LC-PUFAs (arachidonic acid, ARA; and docosahexaenoic acid, DHA) are elevated in serum from AfAm relative to EuAm from the same clinical diabetes study cohort [106].

Similarly, studies over the past decade show that diverse global populations have differences in their capacity to utilize 18C-PUFAs to synthesize LC-PUFAs, and there is now strong evidence that common *FADS* variants form ancestral and derived haplotypes that account for these pathway efficiency differences. It has been recently proposed that the derived haplotype played a crucial role in human evolution under circumstances when dietary LC-PUFAs, especially dietary *n*-3 LC-PUFA levels, were low [119,131,132,133]. This included movement away from *n*-3 LC-PUFA-rich marine sources during the ‘great expansion’ in Africa 60,000–80,000 years ago and the adaptation to largely vegetarian diets after the development of agriculture in Europe and Asia ~12,000 years ago. Consequently, African, African ancestry and some south Asian populations have high frequencies of a derived haplotype that is associated with efficient LC-PUFA biosynthesis [117,119]. Additionally, the MWD provides very high levels of LA to this genetically more efficient pathway resulting in significantly higher levels of the *n*-6 LC-PUFA, ARA, when compared to most European or European ancestry populations [100,106]. Figure 4 illustrates this point by demonstrating that there are significant differences between circulating LC-PUFAs, ARA and DHA, in African and European American populations. A major question that arises from the data in Figure 4 is; what are the biological consequences and specifically the risk of human disease for individuals who are either at the upper (excess ARA levels) or lower (depressed DHA levels) end extremes?

Numerous studies have addressed the issue of excess ARA or efficient LA to ARA conversion in the context of cardiovascular disease (CVD). For example, Martinelli and colleagues [116] examined associations among 13 *FADS* genotypes, desaturase activity (as determined by ARA/LA ratios), inflammation (C-reactive protein (CRP)), and coronary artery disease (CAD) in 876 individuals with (*n* = 610) and without (*n* = 266) CAD. Individuals carrying certain haplotypes (the derived haplotype) had higher ARA/LA ratios in red blood cell membranes, corresponding to enhanced desaturase activity. Importantly, the ARA/LA ratio was an independent risk factor for CAD (Odds Ratio 2.55, *p* < 0.001), Furthermore, the pro-inflammatory marker, CRP increased progressively across tertiles of ARA/LA. This study provided a powerful example of how variants in the *FADS* cluster alter molecular phenotypes, which in turn alter disease risk. Li and colleagues also examined the association of *FADS* genotypes and plasma fatty acids in control (*n* = 510) and CAD patients (*n* = 505) from a Chinese Han population [111]. They also showed that the ARA/LA ratio was higher in CAD patients and the low pathway efficiency T allele at rs174537 was associated with a lower risk of CAD (Odds Ratio 0.74, *p* = 0.001). Similarly, Kwak and colleagues [105] carried out a case-control study in a Korean cohort and discovered that minor T allele at rs174537 was associated with lower risk of CAD (Odds Ratio 0.75, *p* = 0.006), and T allele carriers had significantly lower pathway efficiency as measured by ARA/LA and ARA/DGLA ratios. The T allele was also associated with lower total-and LDL-cholesterol and lipid peroxides. Importantly, Lettre and colleagues performed a meta-analysis on five African American cohorts (*n* ~ 8,000) and confirmed the association of *FADS* SNPs with not only lipid phenotypes, but CAD itself [137]*.*

Other studies have demonstrated that high levels of ARA in adipose tissue are associated with elevated risk of acute myocardial infarction (AMI) [138,139]. For example, Kark and colleagues showed that ARA in adipose tissue was positively associated with AMI (O.R. 2.12, *p* = 0.004). Our laboratory has demonstrated that the genotypes at the *FADS1* SNP, rs174537, which increases the efficiency of the LC-PUFA biosynthetic pathway, and thus circulating and cellular ARA levels, are also associated with higher levels of pro-inflammatory ARA-derived eicosanoids [140]. ARA-derived eicosanoids, such as urinary 8 epi-prostaglandin F(2α) that are independent risk factors for CAD, are also positively correlated with levels of ARA and ARA /LA ratios [105,141]. Together, these studies suggest that individuals and populations that have an enhanced genetic capacity to convert high levels of dietary LA to ARA are more likely to have high levels of ARA, ARA metabolites, inflammatory biomarkers, and diseases of inflammation such as CAD.

Although the aforementioned findings show genetic variation in the *FADS* cluster to be cross-sectionally associated with LC-PUFA and LC-PUFA metabolite levels, biomarkers of inflammation and CAD risk, few studies have investigated whether genetic variation within the *FADS* cluster is a significant mediator of the relationship between PUFA intake and CVD risk. One longitudinal cohort study [142] with a mean follow-up of 14 years, which included 24,032 participants aged 44–74 years, reported a borderline significant interaction by genotype of the *FADS* SNP rs174546 on the incidence of CVD by PUFA intake levels. Particularly, the ALA-to-LA intake ratio was inversely associated with CVD risk only among participants homozygous for the T-allele of *FADS* SNP rs174546 (HR for quintile 5 vs. quintile 1 = 0.72; 95% CI: 0.50, 1.04; *p*-trend = 0.049). Additionally, ALA intake inversely associated with ischemic stroke only among rs174546 TT genotype carriers (HR for quintile 5 vs. quintile 1 = 0.50; 95% CI: 0.27, 0.94; *p*-trend = 0.02). This study provides some evidence, albeit weak, that genetic variation in the *FADS* cluster may mediate the associations between PUFA intake and CVD risk, and that high ALA intake and a high ALA-to-LA intake ratio may be preferable to help prevent CVD and ischemic stroke particularly among those that are homozygous for the minor T-allele of rs174546. In summary, accumulating evidence suggests that the *FADS* locus may be useful in stratification and targeting of LC-PUFA recommendations for prevention of CVD; however, further research is necessary to better understand how genetic variation within the *FADS* cluster modifies the relationship between PUFA intake and CVD risk [143].

What about individuals/populations with low levels of LC-PUFAs such as DHA? This would be expected in those individuals/populations with *FADS* variants that make up the ancestral haplotype associated with inefficient LC-PUFA biosynthesis. As discussed above, the ancestral haplotype appears to have played a crucial role in the adaptation of Arctic populations to cold environments under conditions where high levels of preformed *n*-3 LC-PUFAs were ingested from the abundant marine sources [129]. Importantly, very high frequencies of this ancestral haplotype are also observed in Native American populations [118,130]. This genetic architecture, together with the current levels of LA, ALA, preformed *n*-3 LC-PUFAs, and specifically low levels of DHA in the MWD raises vital questions of how modern populations with high Native American ancestry acquire DHA. Does the near fixation of the ancestral haplotype, with its limited capacity to synthesize LC-PUFAs in Native American ancestry individuals, together with the PUFA composition (high levels of LA and low levels of ALA and DHA) give rise to *n*-3 LC-PUFA deficiencies and resulting diseases/disorders in Native American populations? DHA is critical for brain function throughout the human life span, but its accumulation is especially important to healthy brain development during gestation and infancy [144,145,146]. Additionally, as described above, *n*-3 LC-PUFAs such as DHA, DPA and EPA and their metabolites have potent anti-inflammatory properties [47,58]. There are no studies to date that have compared circulating and cellular levels of LC-PUFAs in a Native American ancestry cohort to other human populations. Future studies will be necessary to determine the risk of *n*-3 LC-PUFA deficiency in this population and whether *n*-3 LC-PUFA supplementation could provide important health benefits to Native American ancestry populations.

Most European and Asian ancestry populations are polymorphic for derived and ancestral haplotypes and individual genetic variants within the *FADS* cluster that impact LC-PUFA biosynthesis. Consequently, these populations appear to be more diverse with respect to their capacity to synthesize LC-PUFAs. For example, we have demonstrated in three European ancestry cohorts that ~45% of individuals have the homozygous GG genotype at the *FADS1* SNP rs174537, which is associated with efficient LC-PUFA biosynthesis [36,97,100,106]. The TT genotype, which is associated with low efficiency LC-PUFA biosynthesis, is found in ~11% of the individuals in these cohorts and the balance (~44%) have the GT genotype. Similar to modern Native American ancestry populations, these studies raise important questions of how the 11% of individuals of European ancestry with the TT genotype and thus less efficient LC-PUFA biosynthesis acquire *n*-3 LC-PUFAs and particularly DHA when they are consuming a MWD.

Studies are just now beginning to emerge that indicate that epigenetics and specifically the methylation status of specific CpG sites within the *FADS* cluster (in a regulatory region between *FADS1* and *FADS2* that contains the *FADS1* and *FADS2* promoters and an regulatory enhancer) impacts the transcription of *FADS* cluster genes, LC-PUFA biosynthesis, and in one study, both immediate and delayed memory performance in toddlers [147,148]. A separate human study shows that previous PUFA exposure impacts the methylation status of GpG sites within this region [149]. We performed genome-wide allele-specific methylation (ASM) with the *FADS1* SNP, rs174537 in 144 human liver samples and identified highly significant ASM with CpG sites between *FADS1* and *FADS2* in a enhancer signature region [150], leading to the hypothesis that the associations of rs174537 with LC-PUFA levels may be impacted by the methylation status of that enhancer region. Additionally, a study in rats indicates that maternal fat intake alters ARA and DHA status and the epigenetic regulation of *FADS2* in offspring liver [151]. Although these studies are still early, collectively, they suggest that there are likely to be factors such as age, sex, pregnancy and prior PUFA exposure that impact epigenetic-mediated regulatory mechanisms of the *FADS* cluster leading to alterations in *FADS* gene transcription, LC-PUFA biosynthesis from 18C-PUFAs and ultimately LC-PUFA status [149]. Understanding the ramifications of these epigenetic modifications will likely be important in discerning how LC-PUFA levels are regulated both at individual and population levels.

## 6. Implications of Non-Uniform *n*-3 LC-PUFA Biosynthesis on the Efficacy of *n*-3 LC-PUFA Supplementation Trials

Pioneering studies 40 years ago showed that high dietary intake of *n*-3 LC-PUFA-enriched foods reduced mortality from myocardial infarction in Greenland Eskimos [152,153]. Since then, a growing body of evidence has shown that dietary *n*-3 LC-PUFAs may impact cardiovascular disease by numerous mechanisms including reducing circulating triglyceride concentrations, inflammatory processes, platelet aggregation and the incidence of arrhythmias and improving endothelial cell function [154]. Studies from predominantly secondary prevention trials and meta-analyses of observational studies in the 1980s through the early 2000s demonstrated a cardioprotective effect of fish consumption and *n*-3 PUFA supplementation [155,156,157,158,159,160]. Additionally, high circulating and dietary levels of *n*-3 LC-PUFAs were shown to associated with lower total mortality, especially deaths due to coronary artery disease [161,162]. However, more recent RCTs and meta-analyses indicate that supplementation with *n*-3 LC-PUFAs is not associated with lower risk of adverse outcomes such as all-cause mortality, cardiac death, sudden death, myocardial infarction, or stroke [163,164,165,166,167].

Similarly, several studies have shown associations between low levels of DHA and/or altered ratios of *n*-6 to *n*-3 LC-PUFAs and cognitive function (memory and Alzheimer’s disease) as well as psychological disorders (attention-deficit/hyperactivity-ADHD, schizophrenia, autism spectrum and major depressive disorders) in children, adolescents or adults [168,169,170,171,172,173,174,175,176,177,178,179,180]. However, systematic reviews and meta-analyses reveal that RCTs show inconsistent results for the therapeutic benefit of *n*-3 LC-PUFAs [181,182,183,184,185,186,187]. This pattern of erratic clinical results with *n*-3 LC-PUFAs is also observed in several inflammatory diseases including asthma [188,189], rheumatoid arthritis [190,191] and cancer [192,193,194,195].

Together, these studies and the controversies stemming from them have led to great confusion among clinicians and consumers alike about the efficacy of *n*-3 LC-PUFAs for the prevention and treatment of human disease. These varying clinical results have been particularly difficult to comprehend in light of the vast numbers of studies that have examined the mechanism by which *n*-3 LC-PUFAs exert their effects and convincing in vivo data with LC-PUFAs in animal models. Recently, experts at the International Society for the Study of Fatty Acids and Lipids discussed experimental design issues that may contribute to inconsistent results with *n*-3 LC-PUFA interventions in cardiovascular studies and thus must be considered in future clinical designs [196]. These included: (1) the potential of current CVD drug treatments to hide *n*-3 LC-PUFA benefits; (2) the potential impact of high background intakes of LC-PUFAs; (3) small sample sizes; (4) short treatment durations; (5) insufficient dosages of *n*-3 LC-PUFAs; (6) increase in *n*-6 PUFA intake; and (7) failure to measure baseline *n*-3 status. This current review has emphasized the potential impact of the latter three.

First, as discussed in detail above, the dramatic increase in dietary levels of the *n*-6 18C-PUFA, LA, in the MWD has resulted in an imbalance of LA and ALA entering the LC-PUFA biosynthetic pathway (Figure 1). Because these 18C-PUFAs compete for the desaturase and elongase steps in the pathway, the imbalance in LA and ALA together with the limited capacity of the pathway results in a disproportionate synthesis of *n*-6 LC-PUFAs at the expense of *n*-3 LC-PUFAs. The other major consideration is the overall capacity of the pathway to synthesize LC-PUFAs, and a large body of evidence (summarized above) indicates that this pathway capacity is variable in individuals and diverse populations and strongly linked to ancestry. For example, African, African ancestry and most south Asian populations contain evolutionary-driven genetic variants, particularly in the *FADS* cluster, that are strongly associated with efficient LC-PUFA biosynthesis and thus high levels of LC-PUFAs and particularly ARA. We have demonstrated that genetic variants associated with elevated levels of ARA are also associated with the capacity to generate high concentrations of pro-inflammatory eicosanoids in whole blood [140]. Consequently, we hypothesize that the combination of a marked increase in dietary LA as a result of the MWD, together with an enhanced capacity to convert that LA to ARA gives rise to elevated levels of a diverse family of ARA-derived mediators, which promote obesity, inflammation and related diseases. In this case, it may be the excess in ARA and ARA-derived mediators, created by the aforementioned gene–diet interaction, that limits the capacity of *n*-3 LC-PUFAs to impact inflammatory diseases such as CVD and cancer.

On the other hand, individuals of Arctic and Native American ancestry as well as a significant percentage of European and Asian populations have evolutionary-driven *FADS* variants associated with a less efficient LC-PUFA biosynthetic pathway [130]. In this case, we postulate that high levels of LA (relative to ALA) in the MWD are converted to sufficient levels of *n*-6 LC-PUFAs such as ARA and ARA metabolites. However, because of the constraints of the less efficient LC-PUFA biosynthetic pathway, it would be predicted that low quantities of *n*-3 LC-PUFAs and specifically DHA and DHA metabolites would be generated from dietary ALA. Such gene–diet interactions in these individuals/populations could lead to *n*-3 LC-PUFA deficiency, and like other nutrient deficiencies, these may be the patients that most benefit from supplementation with *n*-3 LC-PUFAs. It is important to emphasize that this is a hypothetical scenario at this point in time, and future studies will be necessary to determine actual levels of *n*-3 LC-PUFAs in individuals/populations with the ancestral *FADS* haplotype.

Superko and colleagues reviewed clinical investigations that actually measured blood or plasma levels of *n*-3 LC-PUFAs [197]. Not surprising, their data suggest that diet and geography play a critical role in levels of *n*-3 LC-PUFAs. For example, the lowest 5th percentile of Japanese living in Japan had higher levels of *n*-3 LC-PUFAs than whites living in Pennsylvania and Japanese Americans living in Honolulu. In an analysis of nine studies, Hawkey and Nigg found lower overall blood levels of the *n*-3 LC-PUFAs, EPA and DHA, in individuals with ADHD versus controls [183]. They suggest that there may be “a disruption in the conversion process from ALA to EPA/DHA in the ADHD population”. Several GWAS combined with metabolomics analyses have now examined associations between *FADS* variants and circulating levels of *n*-3 LC-PUFAs. Table 1 shows that *FADS1* variants (rs174547, rs174537, and rs174548) are strongly associated with *n*-3 LC-PUFAs, EPA, DPA or DHA either as free fatty acids or in complex lipids [198,199,200] and further suggest that much of the variation in background *n*-3 LC-PUFA levels is due to genetic variation within the *FADS* cluster.

In addition to blood levels, the review by Superko and colleagues also pointed out that there are marked differences in the impact of *n*-3 LC-PUFA supplementation on circulating levels of LC-PUFAs and altering ratios of *n*-3 to *n*-6 LC-PUFAs [197]. Consequently, large diverse RCTs typically have sizeable subsets of individuals with high, intermediate, and low blood levels of LC-PUFAs; and the degree to which these levels are altered appears highly variable. Thus, it is little wonder that RCTs with *n*-3 LC-PUFAs have yielded perplexing results. Providing individuals with *n*-3 LC-PUFA supplements who do not have LC-PUFA deficiencies, or diverse groups of individuals where diet–gene interactions have created markedly different *n*-6 to *n*-3 LC-PUFA levels and ratios is unlikely to provide clear results. Given the genetic diversity in the LC-PUFA biosynthetic pathway, it may be too much to expect that supplementation strategies that fail to stratify participants in some manner (ex, genetics, background *n*-3 LC-PUFA levels, or ratio of *n*-6 LC-PUFAs to *n*-3 LC-PUFAs) will show statistical efficacy for complex human diseases.

## 7. Conclusions

A primary goal of the emerging field of precision nutrition is to have the capacity to predict physiological and pathological outcomes of human diets based on a better understanding of interacting parameters such as an individual’s genetic capacity to utilize/metabolize certain dietary nutrients and that same individual’s dietary nutrient exposure. Insights gained from such studies also offer the potential to tailor nutritional recommendations and interventions to individuals and populations throughout the life span. The *FADS* cluster and *ELOVL2/5* genes are highly polymorphic, with considerable ancestry-based variation in the frequencies of common variants. These variants are associated with different conversion efficiencies of ALA and LA to *n*-3 LC-PUFAs and *n*-6 LC-PUFAs, respectively, and also important molecular and clinical phenotypes linking them to the pathogenesis of numerous human diseases. Additionally, the imbalance of LA and ALA in the MWD alone has been demonstrated to induce higher levels of *n*-6 LC-PUFA relative *n*-3 LC-PUFAs in circulation, cells and tissues. Considering the high heritability of LC-PUFA biosynthetic capacity (i.e., the strong genetic regulation), marked epigenetic regulation (i.e., potential gene environment interplay in regulation) and differences in dietary PUFA intake (i.e., variability in the environment), it is highly likely that *n*-3 LC-PUFA supplementation efficacy is individualized with a complex interplay of genetics, epigenetics and environment.

What are the implications of all this genetic and dietary complexity from a practical application perspective? It is clear that there have been incredible increases in consumption of LA-containing oils, such as soybean oil, in the evolution of the MWD over the past 75 years, and that these dietary changes have led to dramatic increases in LA/ALA ratios and ARA-derived metabolites, and reductions in both circulating and tissue levels of *n*-3 LC-PUFAs. This has occurred during a time of marked increases in obesity and obesity-related inflammatory diseases. Indeed, the concept of ‘Omega-3 Deficiency Syndrome’ introduced by Okuyama and colleagues [84] as Japanese populations moved from their native to a Western diet, may apply to most individuals exposed to the MWD. Consequently, we believe it is safe to say that a reduction in the dietary intake of LA and ARA, together with an increase in *n*-3 LC-PUFAs would benefit most individuals. However, the fact that there are such individual- and population-based genetic differences in the metabolism of dietary 18C-PUFAs, resulting in high, intermediate, and low blood levels of *n*-6 and *n*-3 LC-PUFAs and LC-PUFA metabolites, suggests that some populations and individuals will respond to *n*-3 LC-PUFA supplementation better than others. Based on this premise, it is important for future investigations that focus on *n*-3 LC-PUFA supplementation for the prevention and management of human diseases to develop therapeutic strategies taking into consideration the genetic heterogeneity in their study populations.

## Figures and Tables

**Table 1:** Association of key FADS SNPS with serum LC-PUFAs.

	SNPs			
*n*-3 PUFA/Metabolite	rs174547 *p*-Value	rs174537 *p*-Value	rs174548 *p*-Value	Data Source
1-hexadecanoyl-2-docosapentaenoyl-GPC (16:0/22:5*n*3)	2.97 × 10^−95^	3.83 × 10^−93^	9.17 × 10^−88^	Draisma et al. [198]
1-tetradecanoyl-2-docosapentaenoyl-GPC (14:0/22:5*n*3)	2.76 × 10^−58^	1.94 × 10^−57^	5.08 × 10^−54^	Draisma et al. [198]
1-octadecanoyl-2-docosapentaenoyl-GPC (18:0/22:5*n*-3)	2.23 × 10^−42^	4.61 × 10^−41^	2.53 × 10^−40^	Draisma et al. [198]
1-*O*-docosanyl-2-docosahexaenoyl-GPC (o-22:0/22:6*n*-3)	1.67 × 10^−40^	6.03 × 10^−40^	3.15 × 10^−37^	Draisma et al. [198]
1-*O*-hexadecyl-2-docosahexaenoyl-GPC (o-16:0/22:6*n*-3)	1.35 × 10^−25^	4.90 × 10^−24^	1.36 × 10^−23^	Draisma et al. [198]
1-eicosanoyl-2-docosahexaenoyl-GPC (20:0/22:6*n*-3)			2.19 × 10^−23^	Draisma et al. [198]
eicosapentaenoate (EPA; 20:5*n*3)	1.12 × 10^−21^	2.55 × 10^−21^	3.71 × 10^−22^	Shin et al. [199]
1-octadecanoyl-2-docosahexaenoyl-GPC (18:0/22:6*n*-3)	8.48 × 10^−20^	3.88 × 10^−19^	1.70 × 10^−18^	Draisma et al. [198]
1-tetradecanoyl-2-docosahexaenoyl-GPC (14:0/22:6*n*-3)	9.86 × 10^−18^	2.72 × 10^−17^	1.38 × 10^−15^	Draisma et al. [198]
1-octadecanoyl-2-docosapentaenoyl-GPC (18:0/22:5*n*3)	4.43 × 10^−14^	9.83 × 10^−14^	9.99 × 10^−16^	Long et al. [200]
octadecatetraenoic acid (stearidonate) (18:4*n*3)	1.63 × 10^−15^	1.07 × 10^−15^	1.97 × 10^−13^	Shin et al. [199]
1-*O*-octadecyl-2-docosahexaenoyl-GPC (o-18:0/22:6*n*-3)	2.02 × 10^−15^	5.04 × 10^−15^	1.47 × 10^−14^	Draisma et al. [198]
1-hexadecanoyl-2-docosahexaenoyl-GPC 16:0/22:6	1.21 × 10^−14^	4.16 × 10^−14^	3.02 × 10^−13^	Draisma et al. [198]
1-eicosatrienoyl-GPC (ETA; 20:3*n*-3)			1.30 × 10^−14^	Shin et al. [199]
docosapentaenoate (DPA; 22:5*n*-3)	2.93 × 10^−13^	5.07 × 10^−13^		Shin et al. [199]
1-eicosapentaenoyl-GPC (20:5*n*-3)			1.59 × 10^−12^	Long et al. [200]
1-octadecenoyl-2-eicosapentaenoyl-GPC (18:1/20:5*n*-3)			1.78 × 10^−12^	Long et al. [200]
1-palmitoyl-2-eicosapentaenoyl-GPC (16:0/20:5*n*-3)			1.01 × 10^−11^	Long et al. [200]

Association of three key *FADS* SNPS with levels of serum *n*-3 LC-PUFAs either as free fatty acids or esterified complex lipids. Serum levels of *n*-3 LC-PUFAs and glycerol-3-phosphocholine (GPC) containing *n*-3 LC-PUFAs were associated with three SNPS in *FADS1* gene region. Data are derived from SNiPA analysis [201] of studies by Draisma et al. [198]; Long et al. [200]; and Shin et al. [199]. Significant associations are shown for Bonferroni adjusted *p*-values for each of three representative studies.
